# Concerns and coping mechanisms of breast cancer survivor women from Asia: a scoping review

**DOI:** 10.1007/s00520-023-07996-w

**Published:** 2023-08-19

**Authors:** Bhakti Sarang, Prashant Bhandarkar, Shradha S. Parsekar, Priti Patil, Jubina Balan Venghateri, Rakhi Ghoshal, Deepa Kizhakke Veetil, Priyansh Shah, Anita Gadgil, Nobhojit Roy

**Affiliations:** 1 WHO Collaborating Centre for Research in Surgical Care Delivery in LMICs, Mumbai, India; 2 Department of Surgery, Terna Medical College & Hospital, New Mumbai, India; 3 Department of Statistics, WHO Collaborating Centre for Research on Surgical Care Delivery in LMICs, BARC Hospital, Mumbai, India; 4 Tata Institute of Social Sciences, Mumbai, India; 5 Public Health Evidence South Asia, Prasanna School of Public Health, Manipal Academy of Higher Education, Manipal, Karnataka India; 6 CARE India, Patna, Bihar India; 7 Department of Surgery, Manipal Hospital, Delhi, India; 8 Research Affiliate, Department of Global Public Health, Karolinska Institutet, Stockholm, Sweden

**Keywords:** Breast cancer, Survivors, Asia, Women, Concerns, Coping mechanisms, Scoping review, Oncology, Survivorship issues

## Abstract

**Purpose:**

The incidence of breast cancer has increased significantly in Asia due to epidemiological transition and changes in human development indices. Advancement in medical technology has improved prognosis with a resultant increase in survivorship issues. The effects of breast cancer diagnosis and treatment are influenced by the patient’s cultural beliefs and social systems. This scoping review aims to summarise concerns and coping mechanisms of women with breast cancer in Asia and understand gaps in the existing literature.

**Methods:**

We performed a scoping review using the population-concept-context strategy. A systematic search of MEDLINE (PubMed, Web of Science), CINAHL, SCOPUS, and Embase was conducted for studies conducted in Asia on women diagnosed with breast cancer, identifying their concerns and coping mechanisms, published between January 2011 and January 2021. Data from included studies were reported using frequencies and percentages.

**Results:**

We included 163 studies, of which most (81%) were conducted in hospital settings. Emotional and psychological concerns were reported in 80% of studies, followed by physical appearance and body-image concerns in 46%. Social support (59%), emotion-based coping (46%), spirituality, and problem-based coping (37%) were the major coping systems documented.

**Conclusion:**

The mapped literature documented that anxiety, depression, and fear of cancer recurrence dominated women’s emotional concerns. Women coped with the help of social support, positive reappraisal, and faith in God and religion. Sensitization of caregivers, including healthcare professionals and family members, to context-specific concerns and inquiry into the patients’ available support systems is essential in strengthening breast cancer women’s recovery and coping.

**Supplementary Information:**

The online version contains supplementary material available at 10.1007/s00520-023-07996-w.

## Introduction

Breast cancer (BC) is the most commonly diagnosed cancer in women globally. BC accounts for 11.7% of total cancer cases, with an estimated 2.3 million new cases annually globally [1]. The incidence of breast cancer is annually increasing by 4–6% over the past three decades. The burden is higher in Asian countries, especially where epidemiological transition and changes in human development indices are evident [2]. Advancements in medical technology have improved the diagnostic and treatment modalities resulting in an improved prognosis of breast cancer [3]. Improved survival rates have helped in bringing discussions and research on survivor experiences and quality of life to the forefront.

Breast cancer is more than just a physical illness requiring clinical intervention; it affects a woman’s body and mind in complex ways and is, therefore, described as an “emotionally debilitating illness” [4]. The emotional challenges are commonly due to uncertainty and possible threat to life, altered perception of body image by self and society. The social stigma and isolation add to the physical symptoms and the financial burden of prolonged treatment [5]. Literature shows that breast cancer patients deal with these effects and changes due to cancer and treatment in different ways based on their cultural beliefs and prevalent social systems [6–8]. The existing research articles focus on specific concerns like fertility, body image, and psychological issues in isolation. Nevertheless, survivorship and quality-of-life domains, such as physical symptoms, emotional disturbances, and disruption in social and marital relationships, are interdependent. Hence, conducting a holistic overview of how breast cancer affects all these domains of women’s lives is necessary. Currently, available reviews and studies mainly involve women living in high-income countries (HICs) or African Americans [9, 10]. Literature from Asian countries, home to nearly 60% of the world’s population residing in 48 countries, with the rising incidence of breast cancer, is unavailable. This scoping review aims to plug this gap. It argues that in-depth knowledge of the concerns and coping strategies adopted by Asian women is essential in developing relevant information and counseling strategies.

## Methods

### Design and inclusion criteria

This scoping review followed the Joanna Briggs Institute (JBI) methodology. The JBI provides guidance on each section of the scoping review process, including data extraction and presentation of the results. We used the participants-concept-context strategy as guided by Peters et al [11]. We used the “Preferred Reporting Items for Systematic Reviews and Meta-analysis” extension for scoping review studies (PRISMA-ScR) checklist for reporting [12]. We included quantitative and qualitative studies conducted in Asia on breast cancer patients or survivors, addressing the concerns and/ or coping styles to overcome the challenges of treatment and diagnosis. The detailed inclusion and exclusion criteria are listed in Table 1.

**Table 1:** Inclusion and exclusion criteria

Inclusion criteria	Exclusion criteria
Population	
• Studies that considered women (≥ 18 years) diagnosed with breast cancer. • Women could be in any stage of breast cancer trajectory, i.e., newly diagnosed, under treatment (e.g., mastectomy, chemotherapy, radiotherapy, and/or palliative care) or post treatment phases. • Studies are conducted at any point of time from the diagnosis of cancer or any prognostic stage of cancer. • Studies with all types of cancers if they had subgroup analysis for breast cancer. • Studies including breast cancer patients together with their caregivers.	• Studies that included women with other existing life-threatening conditions before a diagnosis of breast cancer. • Studies that included women who were on life support or not able to respond due to severe mental health issues. • Studies involving men diagnosed with breast cancer or treated for it. • Studies that were undertaken among nurses, doctors, or other healthcare professionals. • Studies that recruited caregivers acting as a proxy to respond/participate in the study on behalf of breast cancer women.
Concept	
• Subjective or objective concerns associated with breast cancer and its treatments. • Handling/managing strategies could be any actions taken by the women diagnosed with breast cancer to overcome the concerns. • Cancer-specific survival needs of breast cancer patients • Studies could have used patient-reported or interviewer-administered outcome measures. • There was no restriction on the definition or instrument that used to measure the outcomes. • Qualitative studies were needed to describe the theme/subtheme related to the phenomenon of our interest, i.e., concern or managing strategies.	• Articles addressing outcomes and interventions related to the following: effectiveness of treatment, therapy, or investigations such as anti-cancer investigations and anti-cancer treatment, antioxidant, cancer-control strategies, nuclear medicine imaging techniques. • Breast cancer screening and early detection uptake, breast cancer awareness, breast self-examination • Cancer trends and risk factors associated with the development of breast cancer • Studies concerning the quality of life
Context	
• Studies that were carried out in Asia irrespective of the economic status of the country. • No restriction on the study setting; it could be community-based or institution-based, or rural or urban setting.	• Studies conducted outside Asia on Asian immigrants or Asian origin women • Studies conducted in transcontinental countries such as Turkey, Armenia, Azerbaijan, Georgia, and Kazakhstan were excluded.
Study design	
• Quantitative observational studies and qualitative studies	• Interventional studies, systematic reviews, literature reviews, and other studies involving secondary data analysis • Studies related to the development of models, clinical practice guidelines, questionnaire development • Conference abstracts, commentaries, and editorials

### Information sources and search

We searched MEDLINE (PubMed and Web of Science), SCOPUS, CINAHL, and Embase for literature published between January 2011 and January 2021. Journal articles published in the English language were eligible to be included. We followed a systematic search strategy initially designed for MEDLINE (via PubMed), which was adapted for each database. The search was formed using the combination of Boolean operators AND, OR, and NOT with search terminologies. The search engine-specific strategy used in PubMed has been attached in the supplementary data file.

### Selection of studies

Studies identified from all search engines were clubbed together in EndNote X7 software and duplicates were removed. A distinct number of articles were exported to “Rayyan” (Rayyan Systems. NC), an intelligent-systematic-review online platform. We followed a two-stage screening process, i.e., title/abstract and full-text screening. Based on title/abstract screening, we eliminated articles not under the purview of population-concept-context-study design. Two independent researchers marked their opinion about article inclusion in a blinded manner in Rayyan. Articles for which both reviewers marked as included or excluded were considered accordingly. In case of a conflict between the two reviewers, a third reviewer reviewed the title/abstract, and inclusion was based on the third reviewer’s opinion. Full texts of the articles were retrieved and screened by dividing the records between the authors (independent screening was not performed). The second reviewer randomly screened 10% of the articles. Discrepancy about the decision to exclude was discussed until both reviewers reached a consensus. Additionally, to overcome selection bias, all the team members discussed each excluded study by meeting on a weekly basis.

### Data synthesis and processing

Based on the reviewers’ clinical knowledge and experience, as well as a literature review about the “Population-Concept-Context-Study design,” iteratively, we developed a data-coding file using an Excel spreadsheet. We included physical symptoms, apprehension about physical appearance and body image, emotional and psychological problems, unease and worries about social life, distress in sexual and fertility issues, and financial and work-related concerns. The concerns were categorized based on the literature review and standard questionnaires assessing women’s well-being after cancer treatment [13], considering the main domains of quality of life. The ways women processed and dealt with these were grouped under two broad categories, emotion-based coping, and problem-based coping. We used a theoretical framework based on the Lazarus and Folkman coping model [14–16].

Theoretical framework: the framework by Lazarus and Folkman describes the process of coping when one is exposed to stressors [16]. The primary appraisal after the stressor ascertains whether the situation faced is stressful or not. The secondary appraisal decides the coping strategies based on whether the problem is perceived as “one that can be solved” and leads to “problem-based coping” or “one that does not seem to have solutions” and leads to “emotion-based coping.” They also explained that both coping responses could coexist. We explain the coping strategies of women based on this framework and broadly divide them into problem-focused coping strategies and emotion-focused coping strategies [14, 17].

The data coding and extraction were initially piloted using two studies by each contributing reviewer (and accordingly modified). Consequently, it was driven again by validating the Excel spreadsheet with the help of 10 studies (qualitative and quantitative). The data coding file consisted of citation details, study design, data collection methods, population characteristics such as age, prognostic stage of breast cancer, country, concerns and needs that the breast cancer patients expressed, and their ways to deal with it. The multitude of concerns reflected in each article included in the review was extracted. We did not critically appraise the individual sources of evidence included in this scoping review.

### Synthesis of results

**Fig. 1:**
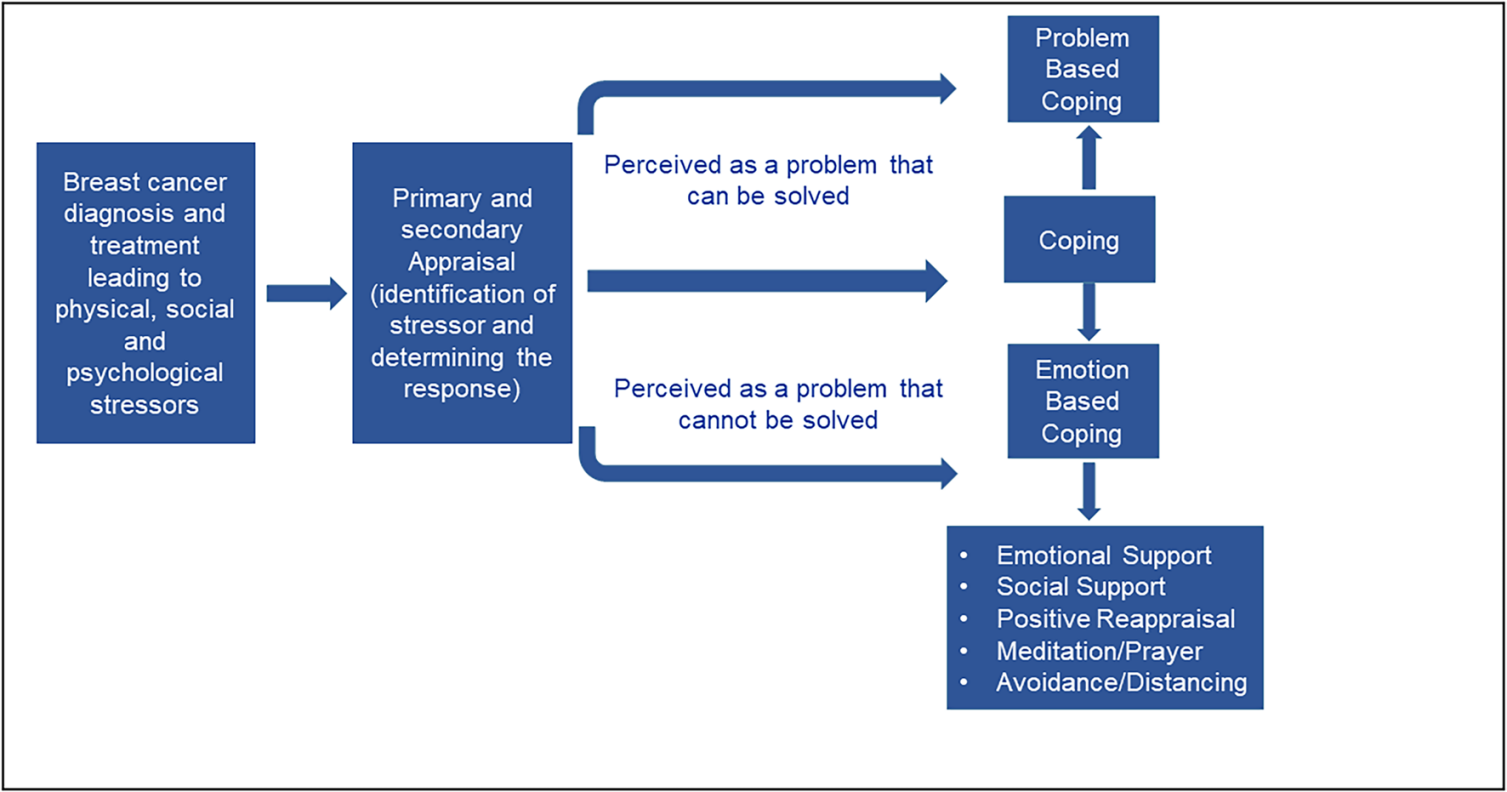
Theoretical framework (ref: Lazarus and Folkman 1984)

Population characteristics among the included studies were mapped across predefined groups and presented accordingly. The data were presented using tables and, wherever appropriate, with figures. The data were described using frequencies and percentages. We classified and tabulated concerns based on broad domains of reported symptoms or stressors and coping strategies by categories based on the theoretical framework (Fig. 1).

## Results

### Study selection

**Fig. 2:**
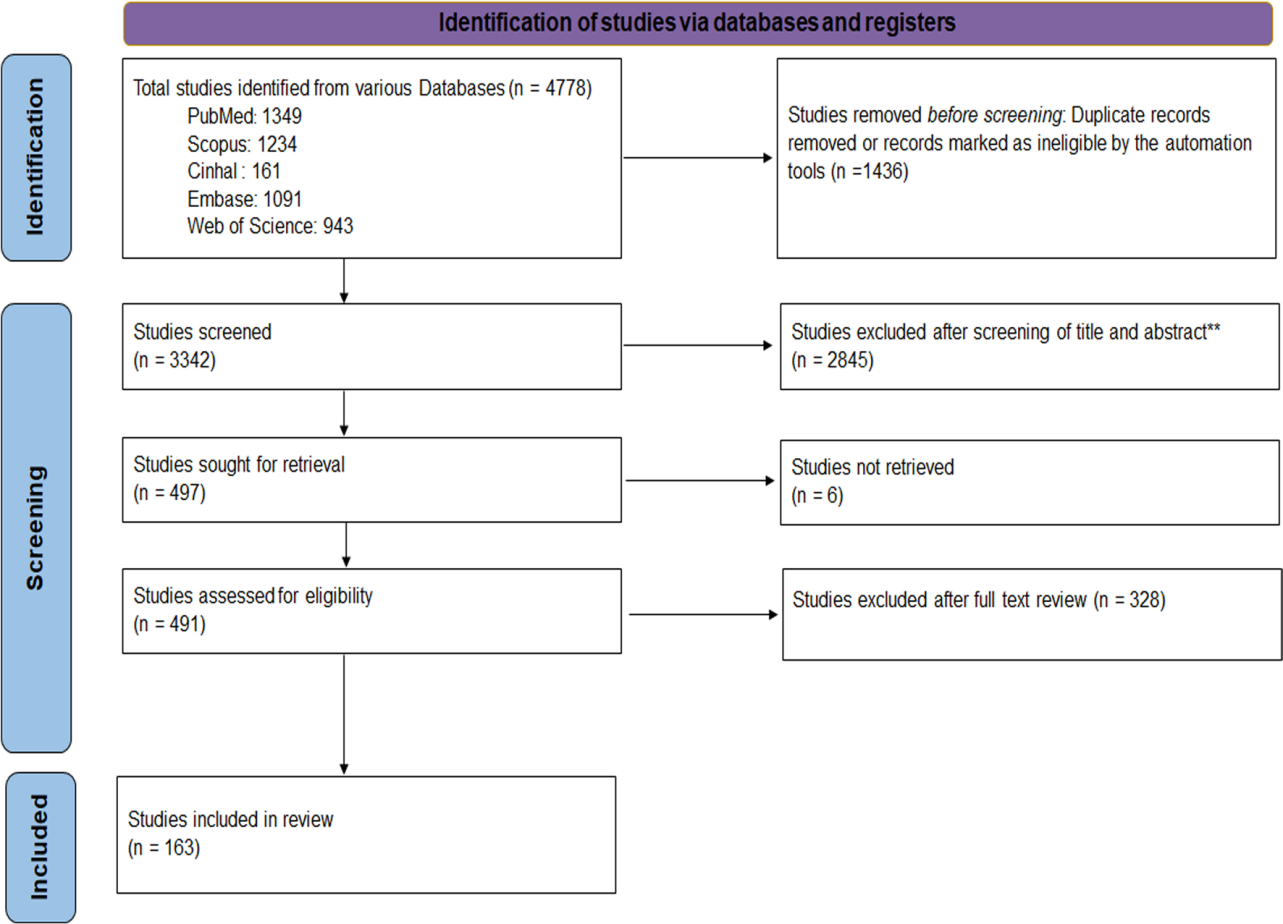
Review process as per PRISMA-2020 flow diagram

Citation hits across various databases generated 3342 records, of which, after title/abstract screening, 497 records were considered for full-text screening. Finally, we included 163 full texts in this review that addressed the concerns and processes of dealing with breast cancer patients and survivors. The detailed study selection process has been documented using PRISMA-2020 (Fig. 2).

### Geographic distribution

**Fig. 3:**
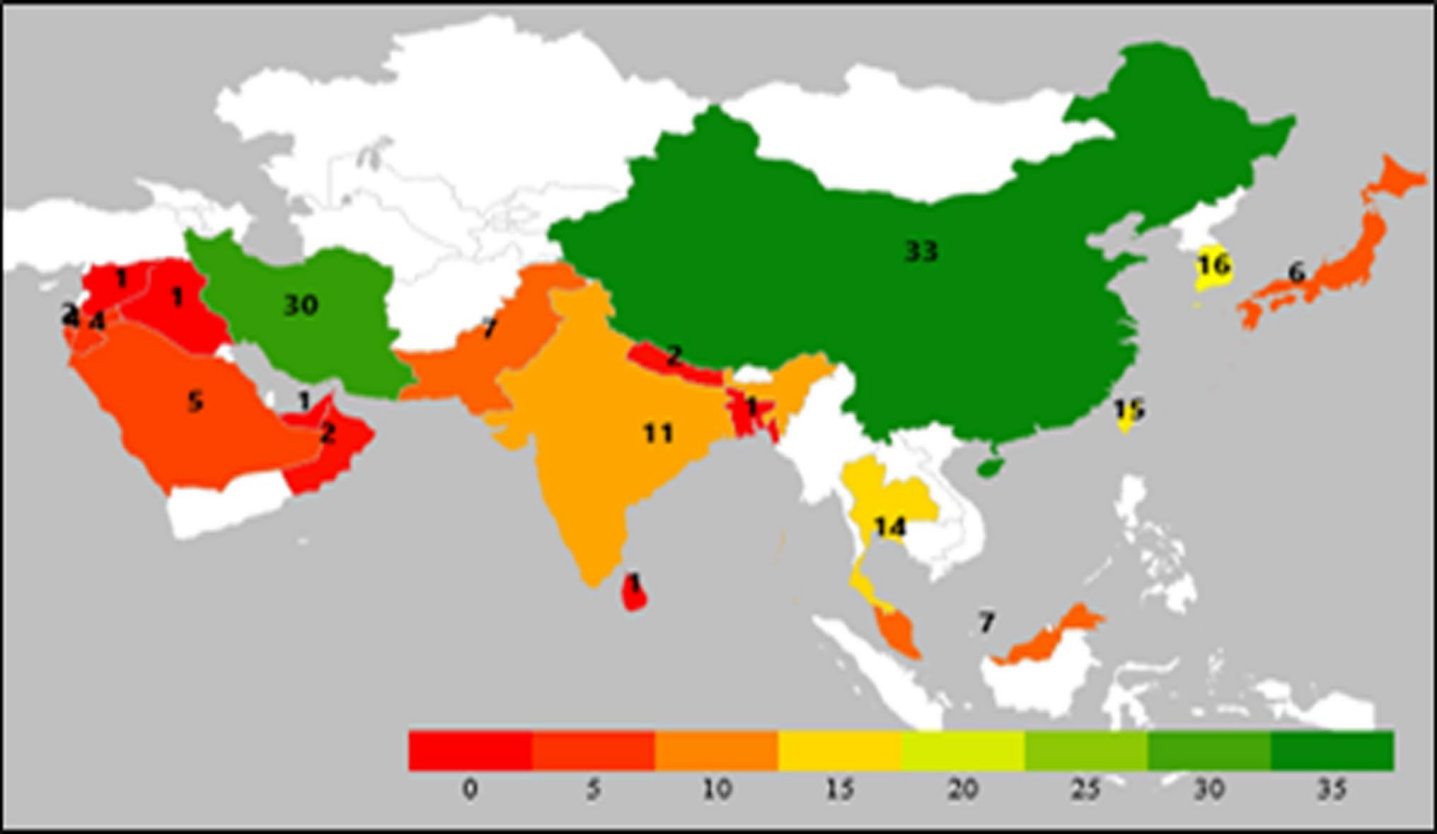
Geographical distribution of the included studies from Asia

Figure 3 shows the geographical distribution of the studies among Asian countries. China, Iran, Taiwan, Thailand, and South Korea contributed 62.5% of the included studies.

### Characteristics of included studies

Table 2 describes the characteristics of the included studies. The majority (80.9%) of the included studies were conducted in the hospital setting during the patients’ hospital visits. Ten (10.5 %) cohort studies addressed the stressors and distress of the women during the treatment and the follow-up period. Of the 163 studies, 127 (77.9 %) used in-depth interviews to collect data from breast cancer survivors.

**Table 2:** Characteristics of the studies included in the review

Description	Number of studies *n* (%)
Study setting	
Hospital-based	132 (80.9)
Community-based	28 (17.2)
Other healthcare facility	3 (1.8)
Study types	
Quantitative studies	95 (58.3)
Cross-sectional	77 (81.0)
Cohort	10 (10.5)
Case-control	4 (4.2)
Not listed	4 (4.2)
Qualitative	59 (36.2)
Mixed methods	9 (5.5)
Cross-sectional	7 (4.3)
Others (not listed = 1, cohort = 1)	2 (1.2)
Sample size	
<10	3 (1.8)
10–49	53 (32.5)
50–99	17 (10.4)
100–499	86 (52.8)
>500	4 (2.5)
Data collection methods	
In-depth/semi-structured interviews	127 (77.9)
Focused-group discussions	3 (1.8)
Other surveys using patient reported outcome measures	33 (20.2)
Data analysis methods	
Thematic	56 (34.4)
Framework	6 (3.7)
Other for quantitative studies	101 (62)

### Population characteristics

The studies included patients with all stages of breast cancer and duration since diagnosis. Ninety-two studies (74.8 %) included women survivors at all stages of cancer. Eight (6.5 %) studies addressed advanced and metastatic cancers. Most (67.6%) of the studies focused on women’s worries and distress in the first year after diagnosis and during treatment, whereas 9% of studies considered long-term concerns of women beyond 5 years from diagnosis. The majority of the studies reported all income and educational groups. None of the studies focused exclusively on single (non-partnered) women, illiterate women, or women from low-income groups (Table 3).

**Table 3:** Study population characteristics

Description	Number of studies *n* (%)
Prognostic stage of breast cancer	123 (75.46)
Stages I and II (early breast cancer)	23 (18.7)
Stages III and IV (advanced/metastatic)	8 (6.5)
Mixed stages	92 (74.8)
Duration of cancer since diagnosis	117 (71.8)
During treatment	47 (40.2)
Up to a year from diagnosis	32 (27.4)
1–5 years from diagnosis	27 (23.1)
>5 years	11 (9.4)
Age groups	146 (89.6)
<35 years (young breast cancer women)	3 (2.1)
35–60 years	11 (7.5)
>60	1 (0.7)
Mixed age groups	131 (89.7)
Education	131 (80.4)
Primary education	8 (6.1)
High school	12 (9.2)
Graduation and above	10 (7.6)
All educational groups	101 (77.1)
Income categories	91(55.8)
All income groups	88 (96.7)
Middle-income group	1 (1.1)
Low-income group	2 (2.2)
Marital status	138 (84.6)
Partnered exclusively	21 (15.29)
Non partnered exclusively	0 (0)
Both groups included	117 (84.8)

### Concerns of breast cancer patients

Table 4 provides the details of concerns, stressors, and challenges experienced by breast cancer patients in Asia. Furthermore, in the section below, we have elaborated on these concerns.

**Table 4:** Concerns of breast cancer patients

Description*	Number of studies *n* (%)
Emotional concerns and problems	131 (80.4)
Anxiety	93 (71)
Sadness/low mood/depression/loneliness	87 (66.4)
Apprehension about future/hopelessness	53 (40.5)
Fear of recurrence of cancer	40 (30.5)
Helplessness/not feeling confident	94 (71.8)
Other concerns like guilt/denial	27 (20.6)
Concerns and perceptions about physical appearance and adverse effects due to symptoms	75 (46.0)
Concerned about adverse effects and inability to work at home due to adverse effects	47 (62.7)
Changes in body symmetry and appearance due to hair loss and breast removal	44 (58.7)
Perception of body-image	33 (44)
Social concerns	58 (35.6)
Social isolation/avoid social and religious gatherings	45 (77.6)
Social stigma/changed reactions and expressions of people	39 (67.2)
Worried about effect of cancer on family	26 (44.8)
Not able to share thoughts with friends/family	16 (27.6)
Physical symptoms	55 (33.7)
Fatigue	40 (72.7)
Pain	34 (61.8)
Nausea and vomiting	26 (47.3)
Insomnia	18 (32.7)
Swelling and lymphedema	17 (30.9)
Sexuality and fertility concerns	36 (22.1)
Decreased interest in sexual life	17 (47.2)
Loss of intimacy with partner	14 (38.9)
Feeling of sexual unattractiveness	13 (36.1)
Other sexual concerns not specified	7 (19.4)
Fertility issues	5 (13.9)
Financial concerns	49 (30.0)
Treatment affordability/lack of insurance	33 (67.3)
Loss or change of work/wages	21 (42.9)
Other concerns	
Lack of information	56 (34.3)

*: Some of the studies reported more than one concern or needs, hence the total does not add up to 100%

#### Emotional and psychological concerns

One hundred thirty-one studies (80.4 %) addressed these concerns. Anxiety, depression, and fear of recurrence were the most common emotional problems seen in breast cancer patients. Included studies documented anxiety and depression in up to 30–39% of women. Women of younger age or those with low socioeconomic status experienced higher psychological distress, whereas strong family support showed beneficial effects in reducing anxiety and loneliness. Ninety-four (71.8 %) studies describing the emotional problems faced by the patients documented that women felt helpless and voiced the need for emotional support. Nine (6.8 %) of these 131 studies described the long-term emotional impact on breast cancer-affected women, whereas 44.2% of studies focused on immediate emotional distress in women less than 1 year from diagnosis.

##### Manifestation of physical symptoms

Out of 163 included studies, 55 (33.7%) addressed physical symptoms and discomforts such as pain, arm swelling, nausea, vomiting, and other symptoms experienced by the women. Three out of 55 (5.4%) studies included physical symptoms experienced after 5 years. Other short-term studies focused on symptoms either during or up to 1 year from treatment. Women also expressed concerns about inadequate information during treatment regarding lymphedema management and the management of side effects of chemotherapy.

##### Concerns about permanent change in physical appearance

Hair loss, swollen arms on the operated side due to lymphedema, and altered body image were reported by 75 (46.0%) studies. Thirty-three (20%) studies described the effect of cancer and its treatment on body image and perception. These concerns were reported more commonly in younger patients and patients undergoing mastectomy. Patients who equated the loss of body parts with “loss of femininity” reported higher psychological distress. Some studies also described that altered body image had adverse effects on the sexual function and the sexual life of patients, but the evidence was not conclusive. Seven (9.3%) out of 75 studies addressed long-term appearance and body image-related apprehension beyond 5 years from diagnosis.

##### Sexual and fertility concerns

These were addressed by 36 (22.1%) studies. Included studies mentioned that up to 40% of women had sexual dysfunction following breast cancer. In addition, some of the included studies dealt with fertility intentions. These studies reported menopausal symptoms as treatment side effects. Younger women wished to continue childbearing and had fertility intentions once the treatment ended.

##### Social concerns

Nearly one-third, i.e., 58 (35.6 %) studies, reported breast cancer women’s concerns related to social aspects of their life. Social isolation and stigma were the two main themes that emerged from our review, addressed by 77% and 67% of studies, respectively. Twenty-six (44.8%) out of 58 studies described that women had apprehension about spreading the disease to other family members.

##### Financial concerns

Of the 163 studies, 49 (30%) described financial concerns. These concerns were mainly related to non-affordability of treatment, loss of wages or jobs due to treatment, poor insurance coverage, and the need for financial support. Fifty-six (34%) studies documented that women were apprehensive and distressed about the lack of adequate information regarding the diagnosis, treatment, and support from healthcare workers. Women needed more assistance and information about the disease and treatment options from healthcare professionals.

##### Trajectories of distress

Longitudinal studies (*n* = 9) tracked the concerns of women through the trajectory of the disease and treatment up to or beyond 1 year or 6 years. These documented low but persistent levels of anxiety, emotional distress, and fatigue for up to 6 years. The studies highlighted that emotional distress, physical symptoms, and fatigue in the first 8–12 months from diagnosis predicted emotional recovery beyond 5 years. Nine (81%) studies in the long-term (more than 5 years from diagnosis) focused on emotional distress in women, whereas long-term sexual and financial concerns were studied by three (27.2%) and four (36%) out of 11 studies, respectively. None of the studies elaborated on the predominance of specific concern over others in a particular recovery phase.

### Coping strategies used by women diagnosed with breast cancer

Out of 163 included studies, 96 (58.8%) focused on women coping with the aforementioned concerns and the strategies involved in dealing with the disease and treatment-related physical and psychological impact. We studied coping strategies using the Lazarus and Folkman theoretical framework. Thirty-six (37.5%) studies highlighted problem-focused management by using information and assistance from the health care system to handle the disease and its side effects and focusing on moving towards a cure. Sixty out of 96 (62.5%) studies addressed emotion-focused coping strategies. Fifty-seven out of 62 (95.0%) studies described women relying on social support through family and friends. Forty-five (75.0%) of the included studies described emotion-based adjustment and dealing with acceptance, finding new meanings and goals in their lives, and even reappraising cancer as a challenge. Thirty-six (60.0%) studies highlighted that women relied on religion or spirituality to help them cope with breast cancer (Table 5).

**Table 5:** Coping strategies used by breast cancer patients

Coping strategies (*n* = 96)*	Number of studies n (%)
Problem-focused coping strategies (obtaining support to cope with side-effects/medical information/ability to manage cancer-related issues)	36 (37.5)
Emotion-focused coping strategies	60 (62.5)
Social-support (family and friends, social support groups, healthcare workers support)	57 (95.0)
Emotion-based management (acceptance, meaning-making, reappraisal of cancer as a challenge or opportunity, positive changes in life)	45 (75.0)
Religious or spirituality/faith in God	36 (60.0)
Physical activity/exercises	11 (18.3)
Meditation/relaxations/mindfulness activity	12 (20.0)
Negative adjustments (denial/avoidance/substance abuse)	6 (10.0)

*: Some of the studies reported more than one coping strategies, hence the total does not add up to 100%

## Discussion

In this review, we compiled the literature from Asia, addressing all the major domains of concerns following breast cancer and women’s ways of dealing with the physical and psychological distress associated with it. The reviewed literature focused on Asian women’s apprehensions about psychological and social disturbances and distress over the changed body image. Using Lazarus and Folkman’s coping theory, we categorized coping strategies as problem-focused and emotion-focused [14–17]. Breast cancer survivors making informed decisions and actively seeking professional help to treat physical symptoms were the main problem-focused coping strategies. Seeking support from family and friends, turning to religion and prayers, diverting attention, and focusing on their roles as caregivers were some of the prominent emotions-focused coping strategies documented in the literature.

### Concerns of the breast cancer patients

#### Emotional concerns

Emotional concern is one of the major problems experienced by women diagnosed with breast cancer. The anxiety and depression persisted much beyond the completion of treatment. Younger women or women with low social support experienced higher emotional disturbance [18]. Wendy et al. quoted a woman saying, “I felt an overwhelming sense of powerlessness and loss of control of life.” This review documented that fear of recurrence dominated in all phases of treatment and beyond. Women described a breast cancer diagnosis as a “death sentence” [19]. However, studies across cultures documented anxiety, depression, and fear of cancer recurrence in breast cancer survivor women. A study documented culture-specific differences between Caucasian and Asian women in anxiety by documenting that the Asian women focused more on the need for information about their disease and treatment. In contrast, Caucasian women emphasized the need for support [20, 21]. The longitudinal studies in our review reflected that overall psychological distress was dependent on and closely followed the physical discomfort during each phase of cancer treatment [22].

##### Physical appearance-related concerns

Perceptions and concerns regarding physical appearance and permanent change in the body were other concerns. Breasts were regarded by women as a “symbol of femininity,” resulting in perceived unattractiveness and lowered self-esteem, and a woman equated “losing one breast to losing a beloved person” [23] [24]. Studies from high-income countries have documented a prevalence of body image concern in as high as 75% of women [25, 26]. Literature has documented that Asian or Asian women living in America were more conservative about discussing sexual health and body-image issues compared to American women [27]. However, contrary to the existing literature, this review adds an important dimension that Asian women were concerned and vocal about body image and sexual disturbances.

##### Sexual concerns

Loss of intimacy with a partner and loss of sexual desire were notable concerns. The studies quoted women saying, “Once afflicted with cancer, the couple should not have sex; otherwise, it will recur. We have not had sex for four years since my diagnosis” [28]. Not prioritizing a woman’s sexual or other needs in a marital relationship could partly be an issue specific to Asian culture, as supported in a study by Marjorie et al [29]. They described cultural differences in perceptions of sexual and marital changes by women and their partners. Authors documented that on direct questioning about the goals of the relationship, Asian couples were more focused on harmony in a marital relationship than intimacy or sexual activity, whereas European-American women focused more on intimacy and physical relationships. Also, when couples of Chinese and Japanese origins were interviewed, the husbands defined the wives as caregivers and self-sacrificing rather than individuals who needed support and love, not prioritizing the needs of the women [29].

##### Social concerns

Women’s worries about changes in social life and isolation were documented by included studies. Women perceived “cancer as a social stigma,” leading them to seek alternative or traditional healing and delay seeking timely medical care [30]. Other reviewed studies concurred with this finding and simultaneously revealed that women expressed a strong social network and support from family and relatives, like spouses, siblings, and parents, during the treatment [19, 31, 32]. This could be attributed to larger and more closely-knit families in the Asian context.

##### Financial concerns

Compared to emotional, social, and body image concerns, financial concerns were less addressed in the reviewed literature. The probable reason could be that traditionally women are not viewed as financial support/bread earners in Asian families, and the males in traditional patriarchal societies carry out that role. Southeast Asia has a large number of low-middle-income countries (LMICs) with high out-of-pocket expenditure, which imposes a huge financial burden on the patients. In this background, more Asian studies are needed to explore the financial burden and its impact on the women undergoing treatment for breast cancer [33].

The lack of information about daily activities, food, exercise, and side effects of treatment was a major theme emerging from the reviewed studies [34]. With access to information on the internet and social media, patients often feel lost in vast pools of information and need the advice of healthcare professionals for clarity [35]. Women expressed that their informed participation in the decision-making and treatment was poor. “Doctor sounded like a recorder, kept on repeating the same information. but when I asked how I can be an active participant in my treatment, he answered that there was nothing I could do” [34].

### Coping strategies in breast cancer patients

This review documents various ways and means cancer patients use to deal with cancer and its effects in all the domains of life. Coping can be defined as an individual’s cognitive or behavioral efforts to manage (decrease or tolerate) situations that are appraised as stress to individuals [36]. The distress management strategies described in this review focused mainly on two categories, problem-solving and emotion-focused coping [14]. The overarching theme that emerged was that women used multifocal strategies, and there was an interplay between different systems and processes to manage some of the cancer-related concerns.

#### Problem-focused coping

Women in the reviewed studies actively adapted lifestyles to face the challenges caused by treatment and its side effects and enhance recovery [37]. Women masked the changes to cope with hair loss and loss of breasts by commonly tattooing eyebrows and wearing scarves over the head and upper body. They also actively participated in the treatment and sought solutions like lymphedema clinics. The Asian women expressed drowning in the flood of information from mass media and other sources and sought active help in solving the problems and choosing the right advice from the information overload. Active problem-solving measures, as per Lazarus and Folkman’s theory [16], are a key to problem-focused coping, as was seen in Asian women.

#### Emotion-focused coping strategies

Social support

The included studies documented that women mainly relied on support from their partners, friends, and family members. The larger or “joint” families that are more prevalent in Asian countries than in the Western culture may have provided a larger pool of caregivers and interactions with social support systems, thus enhancing the role of family and peers in support systems [38]. The women reported a reversal of their caregiving role and expressed receiving emotional and physical support from the family during their treatment [37]. This has also been highlighted by a review by Wellisch et al., where Asian women felt more supported by their families [29]. They were quoted as, “Usually, I’m the one who supports everyone. This time they were very concerned and took care of me” [39]. Although social support was seen to be helpful in most of the reviewed studies, some studies in our review have reported a negative adjustment or avoidance induced by family members’ fears and stigma [40]. The closely-knit families in Asia provide an extensive support network to women and yet can be a cause of concern and fear from social stigma.

Positive reappraisal and meaning-making

Our review documented women positively reappraising the situation and actively working on redefining the meanings and goals of life to cope, described as “meaning making” [14]. The review highlighted that women who focused on minimizing the social disruption or disturbance, e.g., focusing on bringing up children, or another role-functioning, coped better. Women were quoted saying, “I cannot just die, I have a three-year-old child and old parents, I have not fulfilled my obligations to them” [41]. This adds to the insight into culture-specific functional role-driven coping in Asian women. This was also expressed by some of the cross-cultural studies performed in America [7]. The women, as well as their husbands, viewed them as primary caregivers in the family.

Spirituality and religion-based coping: Spirituality and belief in God and religion were common processes to deal with cancer across many religions in Asia, including Buddhism, Islam, and Hinduism [42, 43]. Women found solace and support in the concept of God expressed in the form of prayers and reading of religious scriptures. There was a complex interplay of passive resignation to fate and God, stating they are merely governed by God’s wish, and at the same time, actively seeking support in religious scriptures and God to express themselves and supporting themselves by practicing the spiritual teachings [44]. Women were quoted saying, “I consider this illness a test from God to test my faith. The wisdom is that God still loves me by giving me a chance to be closer to Him”. “Then I would feel that God had granted me peace” [39]*.* Studies across Asia showed consistent evidence that women turned to religion and God to handle stressful situations. This culture-specific coping strategy needs to be considered and highlighted by all the caregivers, especially the healthcare workers, during counseling sessions and patient discussions.

Avoidance coping

Negative adjustment strategies like avoidance, which is categorized as emotion-focused coping by Lazarus and Folkman, were addressed by six out of 96 studies. There was no conclusive evidence of whether avoidance coping helped women to cope better or not. Few studies documented that women who did not accept body changes, used avoidance more prominently and did not cope well [45]. Li et al. reported that women with avoidance and confrontation had better resilience [46]. The authors argued that avoidance distracted women and forced them to focus on other and more positive aspects of their life other than breast cancer. This was under-addressed in our included studies, and it was impossible to derive whether avoidance helped them cope better with cancer. Other behavioral abnormalities such as rage, violence, or substance abuse were not reported as strategies in Asian women.

#### The Asian perspective

This review brings out culture-specific aspects of apprehensions due to breast cancer and dealing strategies to handle these problems. Social isolation and stigma were common worries that Asian women faced leading to delays in diagnosis. Large closely-knit families and relatives were a major source of social support, and women found strength in this network. Women’s role as primary caregivers in Asian families provided an alternative focus for their attention, and during this period of distress, women coped better by positively reappraising the situation and focusing on their functional roles in the family. Thus, family plays an important role in coping with cancer in Asian women.

Spirituality and religion similarly allowed Asian women to accept the disease as “God’s wish” or “testing times given by God.” At the same time, a complete surrender to God is built in the faith that God will help bring an end to suffering and peace.

Contrary to the existing beliefs and literature [23], we documented that Asian women were concerned and vocal about disturbances in their sexual lives and body image distress.

The healthcare workers, as well as other caregivers, need to focus on these culture-specific stressors as well as coping strategies and need to address them from the initial phases of diagnosis and treatment. This review documented that emotional disturbances and unease closely followed physical symptoms, giving key learning for building this factor while attending to physical signs and symptoms.

### Gaps in the literature

This review, as against other studies from Asia which address isolated issues or domains of concerns and strategies of coping among breast cancer women, gives a holistic view of all major domains of distress and strategies to deal with it. In doing so, we highlighted specific gaps in the existing literature. The majority of the reviewed studies were based in the hospital setting. The included studies did not mention the sex or designation and positioning of the interviewing persons. The setting of a clinic and members of the treating team conducting interviews would pose an unequal hierarchical relationship. Also, very few studies addressed the financial and sexual concerns of women. Exclusive studies in women from low socioeconomic status, women with metastatic cancer, and studies addressing end-of-life concerns and issues were very limited.

### Limitations

Although we extensively searched some major databases, this review has limitations. Due to resource constraints, the search was restricted to English language publications, and we did not look at the grey literature and references of included studies. Secondly, we do not refute the possibility of study selection bias, as one researcher reviewed each full text. However, to mitigate this issue, an independent reviewer from the author team reviewed every tenth article for inclusion. Thirdly, there was heterogeneity in the methods and definitions used to describe various concerns and copings. This led to some subjectivity in the data extraction and importance attributed to certain aspects of adjustments and ways of dealing. Nevertheless, we tried to address the reviewers’ subjectivity by having weekly meetings and discussions. We did not assess the critical appraisal of individual sources of evidence.

## Conclusion

Emotional, social (e.g., stigma), and body image-related worries and stressors were predominantly documented in the reviewed literature. Having a close-knit family structure and seeing women as primary caregivers provided a positive reappraisal and helped women cope with social and emotional disturbances. Religion and God played an important role in women coping with cancer. Asian women voiced concerns about body image and sexual disturbances, and these were duly documented in the literature.

## Supplementary information

ESM 1 (PDF 33 kb)

## Data Availability

All the databases that were utilized for this study included open-access articles as well as articles from PubMed, Web of Science, SCOPUS, CINAHL, and Embase. The dataset generated/compiled and/or analysed for this current study has been made available as a supplementary file in the “Supplementary information” section of this submission (data file_Scoping review).

## Abbreviations

LMICs: low-middle-income countries
JBI: Joanna Briggs Institute
PRISMA-ScR: Preferred Reporting Items for Systematic Reviews and Meta-analysis
