# Supplementary material for: Concerns and coping mechanisms of breast cancer survivor women from Asia: a scoping review
Source: Support Care Cancer. 2023 Aug 19;31(9):528. doi: 10.1007/s00520-023-07996-w (PMC10439845; doi:10.1007/s00520-023-07996-w)
Supplement: Supplementary file 1 — (PDF 33 kb) [file 520_2023_7996_MOESM1_ESM.pdf]

Supplementary data

Medline (via PubMed) search strategy

(((((((("China"[Title/Abstract] OR "India"[Title/Abstract] OR "Indonesia"[Title/Abstract] OR "Pakistan"[Title/Abstract] OR "Bangladesh"[Title/Abstract] OR "Japan"[Title/Abstract] OR "Philippines"[Title/Abstract] OR "Vietnam"[Title/Abstract] OR "Turkey"[Title/Abstract] OR "Iran"[Title/Abstract] OR "Thailand"[Title/Abstract] OR "Myanmar"[Title/Abstract] OR "Burma"[Title/Abstract] OR "South Korea"[Title/Abstract] OR "Afghanistan"[Title/Abstract] OR "Iraq"[Title/Abstract] OR "Saudi Arabia"[Title/Abstract] OR "Uzbekistan"[Title/Abstract] OR "Malaysia"[Title/Abstract] OR "Yemen"[Title/Abstract] OR "Nepal"[Title/Abstract] OR "North Korea"[Title/Abstract] OR "Sri Lanka"[Title/Abstract] OR "Kazakhstan"[Title/Abstract] OR "Syria"[Title/Abstract] OR "Cambodia"[Title/Abstract] OR "Jordan"[Title/Abstract] OR "Azerbaijan"[Title/Abstract] OR "United Arab Emirates"[Title/Abstract] OR "UAE"[Title/Abstract] OR "Tajikistan"[Title/Abstract] OR "Israel"[Title/Abstract] OR "Laos"[Title/Abstract] OR "Lebanon"[Title/Abstract] OR "Kyrgyzstan"[Title/Abstract] OR "Turkmenistan"[Title/Abstract] OR "Singapore"[Title/Abstract] OR "Oman"[Title/Abstract] OR "Palestine"[Title/Abstract] OR "Kuwait"[Title/Abstract] OR "Georgia"[Title/Abstract] OR "Mongolia"[Title/Abstract] OR "Armenia"[Title/Abstract] OR "Qatar"[Title/Abstract] OR "Bahrain"[Title/Abstract] OR "Timor-Leste"[Title/Abstract] OR "Cyprus"[Title/Abstract] OR "Bhutan"[Title/Abstract] OR "Maldives"[Title/Abstract] OR "Brunei"[Title/Abstract] OR "Taiwan"[Title/Abstract] OR "Macao"[Title/Abstract] OR "Hong Kong"[Title/Abstract] OR "Asia"[Title/Abstract] OR "south Asia"[Title/Abstract] OR "southeast Asia"[Title/Abstract] OR "south-east Asia"[Title/Abstract]) AND ("Anxiety"[MeSH Terms] OR "anxiety, separation"[MeSH Terms] OR "Anxiety Disorders"[MeSH Terms] OR "phobia, social"[MeSH Terms] OR

"Fear"[MeSH Terms] OR ("Depression"[MeSH Terms] OR "Depressive Disorder"[MeSH Terms] OR "depressive disorder, major"[MeSH Terms]) OR ("Body Image"[MeSH Terms] OR "Body Dysmorphic Disorders"[MeSH Terms] OR "Body Dissatisfaction"[MeSH Terms]) OR ("perception*"[Title/Abstract] OR "need*"[Title/Abstract] OR "stress"[Title/Abstract] OR "distress"[Title/Abstract] OR "hopelessness"[Title/Abstract] OR "apprehension"[Title/Abstract] OR "concern*"[Title/Abstract] OR "worr*"[Title/Abstract] OR "Fear"[Title/Abstract] OR "Anxiety"[Title/Abstract] OR "Depression"[Title/Abstract] OR "Body Image"[Title/Abstract] OR "experience"[Title/Abstract]) OR "Sexuality"[MeSH Terms] OR ("emotion*"[Title/Abstract] OR "psychological"[Title/Abstract] OR "psycho-social"[Title/Abstract] OR "wellbeing"[Title/Abstract] OR "well-being"[Title/Abstract] OR "mental"[Title/Abstract]) OR "adaptation, psychological"[MeSH Major Topic] OR ("coping"[Title/Abstract] OR "adjustment*"[Title/Abstract] OR "personality"[Title/Abstract]) OR "Spirituality"[MeSH Major Topic] OR "Faith Healing"[MeSH Major Topic] OR ("Social Support"[MeSH Terms] OR "Psychosocial Support Systems"[MeSH Terms]) OR "resilience, psychological"[MeSH Major Topic] OR ("family support"[Title/Abstract] OR "caregiver support"[Title/Abstract] OR "community support"[Title/Abstract]) OR "Pastoral Care"[MeSH Major Topic] OR ("religio*"[Title/Abstract] OR "spiritual*"[Title/Abstract]) OR ("attitude"[Title/Abstract] OR "behavior"[Title/Abstract] OR "behaviour"[Title/Abstract]) OR ("satisfaction"[Title/Abstract] OR "Survivorship"[Title/Abstract]) OR "Survivorship"[MeSH Major Topic] OR "Sense of Coherence"[MeSH Major Topic]) AND ("Breast Cancer Lymphedema"[MeSH Major Topic] OR ("Breast Neoplasms"[MeSH Terms] OR "Unilateral Breast Neoplasms"[MeSH Terms] OR "Triple Negative Breast Neoplasms"[MeSH Terms] OR "Inflammatory Breast Neoplasms"[MeSH Terms]) OR (("cancer s"[All Fields] OR "cancerated"[All Fields] OR "canceration"[All Fields] OR "cancerization"[All Fields] OR "cancerized"[All Fields] OR "cancerous"[All Fields] OR "neoplasms"[MeSH Terms] OR

"neoplasms"[All Fields] OR "cancer"[All Fields] OR "cancers"[All Fields] OR ("carcinoma"[MeSH Terms] OR "carcinoma"[All Fields] OR "carcinomas"[All Fields] OR "carcinoma s"[All Fields]) OR ("neoplasm s"[All Fields] OR "neoplasms"[MeSH Terms] OR "neoplasms"[All Fields] OR "neoplasm"[All Fields]) OR "malignan*"[All Fields] OR ("cysts"[MeSH Terms] OR "cysts"[All Fields] OR "cyst"[All Fields] OR "neurofibroma"[MeSH Terms] OR "neurofibroma"[All Fields] OR "neurofibromas"[All Fields] OR "tumor s"[All Fields] OR "tumoral"[All Fields] OR "tumorous"[All Fields] OR "tumour"[All Fields] OR "neoplasms"[MeSH Terms] OR "neoplasms"[All Fields] OR "tumor"[All Fields] OR "tumour s"[All Fields] OR "tumoural"[All Fields] OR "tumourous"[All Fields] OR "tumours"[All Fields] OR "tumors"[All Fields]) OR ("cysts"[MeSH Terms] OR "cysts"[All Fields] OR "cyst"[All Fields] OR "neurofibroma"[MeSH Terms] OR "neurofibroma"[All Fields] OR "neurofibromas"[All Fields] OR "tumor s"[All Fields] OR "tumoral"[All Fields] OR "tumorous"[All Fields] OR "tumour"[All Fields] OR "neoplasms"[MeSH Terms] OR "neoplasms"[All Fields] OR "tumor"[All Fields] OR "tumour s"[All Fields] OR "tumoural"[All Fields] OR "tumourous"[All Fields] OR "tumours"[All Fields] OR "tumors"[All Fields])) AND "english"[Language] AND ("breast"[Title/Abstract] AND "english"[Language]) AND "english"[Language])) AND ("english"[Language] AND 2011/01/01:2021/12/31[Date - Publication])) NOT (("systematic review"[Title/Abstract] OR "review"[Title/Abstract] OR "meta-analysis"[Title/Abstract] OR "meta-analysis"[Title/Abstract] OR "randomised controlled trial"[Title/Abstract] OR "randomized controlled trial"[Title/Abstract] OR "experimental study"[Title/Abstract] OR "clinical trial"[Title/Abstract]) AND "english"[Language])) AND 2010/01/01:2021/12/31[Date - Publication]) NOT ("gene"[Title/Abstract] OR "Genetic"[Title/Abstract] OR "polymorphism"[Title/Abstract] OR "Chromosomes"[Title/Abstract] OR "Pathology"[Title/Abstract])) AND

2010/01/01:2021/12/31[Date - Publication]) NOT "screening"[Title]) NOT "trastuzumab"[Title]) NOT "Immunology"[Title]
